# The Modulatory Effect of Sodium Propionate Treatment in the Expression of Inflammatory Cytokines and Intracellular Growth of *Brucella abortus* 544 in Raw 264.7 Cells

**DOI:** 10.4014/jmb.2303.03041

**Published:** 2023-05-28

**Authors:** Heejin Kim, Tran Xuan Ngoc Huy, Trang Thi Nguyen, Alisha Wehdnesday Bernardo Reyes, WonGi Min, Hu Jang Lee, Jin Hur, Suk Kim

**Affiliations:** 1 Institute of Animal Medicine, College of Veterinary Medicine, Gyeongsang National University, Jinju 52828, Republic of Korea; 2 Department of Veterinary Paraclinical Sciences, College of Veterinary Medicine, University of the Philippines Los Baños, College, Laguna 4031, Philippines; 3 College of Veterinary Medicine, Chonbuk National University, Iksan 54596, Republic of Korea; 4 Institute of Applied Sciences, HUTECH University, 475A Dien Bien Phu St., Ward 25, Binh Thanh District, Ho Chi Minh City 72300, Vietnam

**Keywords:** *Brucella abortus*, sodium propionate, macrophages, cytokines, NF-κB

## Abstract

In this study, we investigated the effects of sodium propionate (SP) treatment on intracellular mechanism of murine macrophages and its contribution to host immunity during *Brucella abortus* 544 infection. The intracellular growth assay revealed that SP inhibited *Brucella* replication inside the macrophages. To determine intracellular signaling involved during SP treatment after *Brucella* infection, we analyzed the change of five different cytokines production relevant to SP such as TNF-α, IL-10, IFN-γ, IL-1β, and IL-6, and the results indicated that the boost with IL-10 was apparent throughout the culture period for 48 h as well as IL-1β which was apparent at 24 h post-infection and IFN-γ which was apparent at 24 h and 48 h in comparison to SP untreated groups. On the other way, SP-treated cells displayed suppressed production of TNF-α and IL-6 at all time points tested and 48 h post-infection, respectively. Furthermore, we conducted western blot to establish a cellular mechanism, and the result suggested that SP treatment attenuated p50 phosphorylation, part of the NF-κB pathway. These findings indicated that the inhibitory effect of SP against *Brucella* infection could be attributed through induction of cytokine production and interference on intracellular pathway, suggesting SP as a potential candidate for treating brucellosis.

## Introduction

*Brucella* are facultative intracellular Gram-negative bacteria that may cause brucellosis in a wide range of different mammals as host and result in enormous economic losses worldwide [1]. Human brucellosis is reported more than 500 thousands new cases with minimal mortality annually and mostly it comes from livestock [2]. Therefore, prevention of the transmission from animal to human and improve incidence in animals can be an effective method to eradicate brucellosis. Vaccination in animal is regarded as one of the prominent ways to control this disease but there is still no vaccine that provides full protection against brucellosis [2]. Understanding the underlying pathogenesis of *Brucella* would be useful in designing effect approaches for the control of the disease in animals and subsequently preventing the disease in human. Once it enters the host, *Brucella* invades phagocytic macrophages and non-phagocytic cells, and it can survive and replicate in the vacuolar phagocytic compartments of macrophages and the endocytic compartments of non-phagocytic cells [3]. *Brucella* has developed its own mechanism to take advantage of the host immune response in order to persist and establish chronic infection and inhibit macrophage apoptosis in infected cells [4, 5]. Furthermore, the infection also encourages macrophages to secrete pro- and anti-inflammatory cytokines such as tumor necrosis factor-α (TNF-α), interleukin-1β (IL-1β), IL-6, interferon-γ (IFN-γ) and IL-10. These immune responses, including the release of ILs, are mediated by NF-κB in part [6]. NF-κB is a central transcription factor responsible for controlling the secretion of multiple cytokine genes involved in inflammatory response [5]. Therefore, the balance between pro-inflammatory and anti-inflammatory cytokine production through NF-κB pathway seems to be important for the host to block chronic infection as well as control survival [4].

Short-chain fatty acids (SCFAs) are carboxylic acids with an aliphatic tail of two to six carbons and are the last products of the fermentation of resistant starch and dietary fibers in the gut [7]. The most abundant SCFAs produced are acetate, propionate, and butyrate, with a molar ratio of 60:20:20 in the intestinal tract. Several studies demonstrated their effect on inflammatory signaling pathways, regulating immune homeostasis, and antimicrobial effect. However, most previous studies mainly concentrated on butyrate, whereas few studies have dedicated their attention to other SCFAs, such as propionate [8]. On that account, the present study focuses on the mechanism of action of sodium propionate (SP) in a macrophage cell line and in a murine model against *Brucella*.

## Materials and Methods

### Bacterial Strains, Cell Culture, and Growth Condition

*B. abortus* 544 (ATCC 2344b), a smooth and biovar 1 strain, was cultured in *Brucella* agar plate and incubated at 37°C for three days to determine the colony forming units (CFUs). One colony gained from agar plate was suspended in *Brucella* broth and incubated at 37°C on a shaking incubator until the stationary phase is reached. Raw 264.7 cell line, the murine macrophage, was cultivated in RPMI 1640: Life Technologies Corporation (USA) with 10% heat-inactivated fetal bovine serum (HI-FBS) with or without 100 U/ml of penicillin and 100 μg/ml of streptomycin (Gibco, Invitrogen, USA) and incubated at 37°C with 5% CO_2_ atmosphere.

### Cell Viability Assay

Raw 264.7 cells were seeded at 4 × 10^4^ cells per well in a 96-well cell culture plate one day before treatment. The SP was prepared using the fresh medium with different concentrations (0.3125, 0.625, 1.25, 2.5, 5, 10, and 20 mM) in a 100 μl volume each well. The cells were washed with PBS once, added 100 μl of new RPMI medium with 10 μl of colorimetric 3-(4,5-dimethlythiazol-2-yl)-2,5-diphenyltetrazolium bromide (MTT) (Amresco, USA) (5 mg/ml) and incubated at least 3 h in dark environment. After incubation, medium was removed and 150 μl of dimethylsulfoxide (DMSO) was added to each well. The absorbance was measured at 540 nm after 15 min. Raw 264.7 cell viability (%) was calculated in comparison with controls that were treated with 0.1% PBS in medium.

### Brucella Survivability Assay

*B. abortus* were diluted using RPMI fresh medium at a concentration of 2 × 10^7^ CFU/ml, and 100 μl was seeded each well in a 96-well plate. Different concentrations of SP (25 and 50 mM) in a 100 μl volume in each well were added and incubated for 0, 2, 24, and 72 h. Each well was diluted in PBS, plated, and incubated at 37°C incubator for 3 days. Bacterial survival (%) was calculated in comparison with controls that were treated with PBS in fresh medium at each time point.

### Bacterial Internalization Assay

*B. abortus* were cultivated in *Brucella* broth and incubated at 37°C at least two days before use. Raw 264.7 cells were seeded in a 96-well plate at 4 × 10^4^ cells per well and incubated at 37°C overnight. For this assay, cells were pretreated with different concentrations of SP (1, 2.5 and 5 mM) for 4 h before infection. After removing the old medium and washing with PBS once, cells were infected with *B. abortus* at a multiplicity of infection (MOI) of 50 in RPMI medium containing 10% HI-FBS, centrifuged at 200 ×*g* for 5 min and incubated at indicated time points. At each time point, cells were washed with PBS, added 100 μg/ml gentamicin in RPMI, and incubated for 30 min to kill extracellular bacteria. After that, cells were washed with PBS again, and 100 μl of distilled water (DW) was added to lyse the cells. Cells were diluted with PBS, plated in a *Brucella* agar plate, and incubated for two days to determine CFUs.

### Bacterial Intracellular Killing Assay

For the intracellular killing assay, Raw 264.7 cells and bacteria were prepared as with internalization assay. Cells were pretreated with three different concentrations of SP (1, 2.5, and 5 mM) 12 h before infection. Bacteria were diluted with medium containing FBS up to 2 × 10^7^ CFU/ml, and cells were infected with 100 μl of diluted bacteria each well at a MOI of 50. After an hour, the medium was changed to a new medium containing SP (1, 2.5, and 5 mM) and gentamicin (100 μg/ml) and incubated at indicated times. Cells were washed with PBS one time, lysed using DW, and plated. Plates were incubated at 37°C for three days to determine CFU.

### RNA Extraction

The total RNA content was isolated from Raw 264.7 macrophages at different time points using a Qiagen RNeasy kit. DNA was eliminated before the final elution of the RNA sample using the Qiagen on-column DNase digestion protocol.

### Measurement of Cytokine Level by qRT-PCR

Real-time PCR analysis was conducted as described previously [6]. Briefly, The SYBR Green PCR master mix (Applied Biosystems, USA) and different pairs of primers (Table 1) were denatured at 95°C for 10 min followed by 40 PCR cycles of 95°C for 15 s, 55°C for 30 s and 60°C for 32 s. The data were analyzed using Bio-Rad CFX software.

### Immune Response Analysis In Vivo

Eight weeks old, female ICR mice were sorted randomly into four groups with eight mice per group (infected with or without SP and non-infected with or without SP) and one week of adaptation preceded all treatment and infection with *B. abortus*. They were maintained in standard condition with ad-libitum food and water. Pre-treatment with SP, the concentration of 50 mg/kg orally, started five days before infection with *B. abortus* via intraperitoneal route at a concentration of 5 × 10^5^ CFUs per mouse. They were sacrificed on the 14^th^ day post-infection, and spleen, liver as well as sera were collected. Each organ was weighed, and 0.05 g was collected and homogenized in PBS. The rest of the samples were kept in formalin for pathological analysis. The homogenates were diluted with PBS 100-fold and spread onto *Brucella* agar plates and then incubated for three days at 37°C.

### Histopathological Assay

Hematoxylin and eosin (H&E) staining was performed on representative lobes of the liver and middle part of the spleen of mice. The ratio of area appearing microgranuloma was measured in specimens of liver and spleen. In addition, the severity of necrosis and periportal inflammation were estimated in liver samples. All the results were scored according to the standard cited from the studies done by Xavier *et al*. [4] and Stranahan *et al*. [9].

### Western Blot Analysis

The procedure of seeding, treating and infection was performed similar to that of intracellular killing assay. Treated and untreated macrophages with or without infection were washed with cold PBS and lysed using cold radioimmunoprecipitation assay (RIPA) buffer with a 1% protease inhibitor cocktail at 4°C for 20 min. Lysed cells were scraped and transferred into a 1.5 ml Eppendorf tube and centrifuged at 12,000 rpm at 4°C for 30 min to collect the lysates. Proteins were denatured by boiling for 5 min in 2 × Laemmli sample buffer mixed with 2-mercaptoethanol. Protein concentrations were measured, followed by the Bradford protein assay (Bio-Rad Laboratories, Inc., USA). An equal protein concentration from each sample was loaded to SDS-PAGE in 10% SDS gel and transferred onto nitrocellulose membrane (Merck Millipore Ltd., Germany) for 20 min using a semi-dry transfer system (Atto Co., Japan). Membranes were incubated with skim milk containing the first antibody of NF-κB P65 (1:200; Santa Cruz, USA), NF-κB P50(1:100; Santa Cruz, USA), and β-actin (1:2000; Cell-Signaling, USA) at 4°C overnight. The membranes were then incubated with secondary antibody using horseradish peroxide (HRP)-conjugated anti-rabbit IgG (1:1000; Cell-Signaling, USA) or HRP-conjugated anti-mouse IgG (1:1000; Promega, USA) for 1 h. After washing with 1X TBS-T, membranes were coated with luminol-coumaric acid-H_2_O_2_ detection solution (Atto Co.) and were exposed to a Molecular Imager ChemiDocTM XRS+ system machine (Bio-Rad Laboratories, Inc.). Protein bands were analyzed using Image Lab software.

### Statistical Analysis

The data of each in vitro experiment were acquired from at least three different experiments with 2 to 6 replicates, and in vivo experimental groups consisted of eight mice expressed as mean ± standard deviation (SD) using GraphPad InStat.

## Results

### Effect of SP on Raw 264.7 Cell Viability and *B. abortus* Survival

**Fig. 1:**
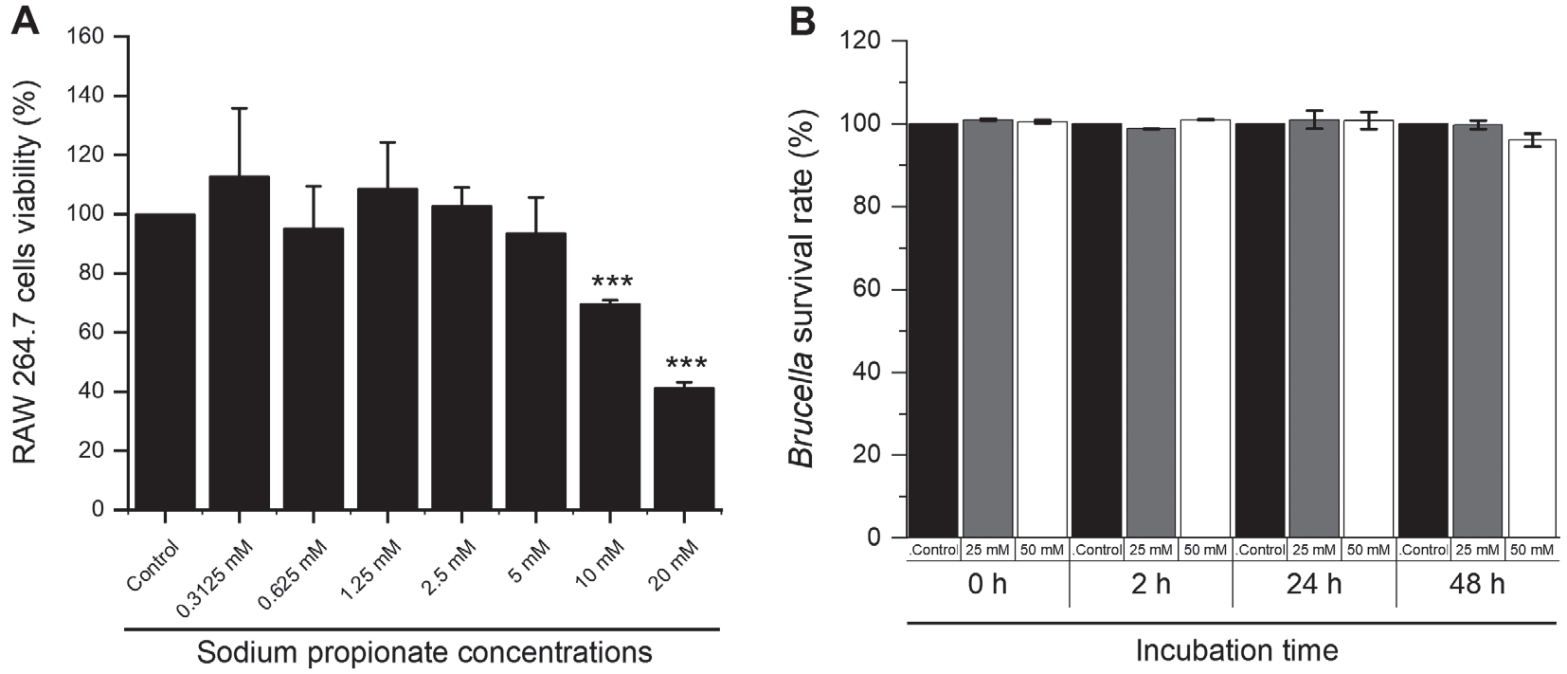
The effect of SP treatment in the viability of macrophages and *Brucella*. Raw 264.7 cells were treated with the different concentrations of SP and the viability was evaluated using MTT assay (**A**). *B. abortus* was incubated with different concentrations of SP for 0, 2, 24 and 48 h and the direct effect was determined (**B**). Data are presented as mean ± SD and statistically differences relative to control group are represented by asterisk (***p < 0.001).

To determine the highest non-toxic concentration of SP in Raw 264.7 cell, different concentrations of SP (0, 0.3125, 0.625, 1.25, 2.5, 5, 10, and 20 mM/ml) were applied to Raw 264.7 cells for 72 h, and the viability was analyzed using MTT assay. We found that from 5 mM and lower concentrations, the viability of the cells was not significantly affected (Fig. 1A). Hence 5, 2.5, and 1.0 mM/ml and lower concentrations were used for succeeding experiments. For the purpose of determining the antibacterial effect of SP on *B. abortus*, *Brucella* was incubated with a medium containing two different concentrations of SP (25 and 50 mM/ml) or 0.1% PBS as the control for 48 h. Comparing the number of CFUs at 48 h post-incubation with control group, both concentrations did not show a significant reduction in bacterial survival (Fig. 1B).

### Effect of SP on Internalization and Intracellular Growth of *B. abortus* in Raw 264.7 Macrophages

**Fig. 2:**
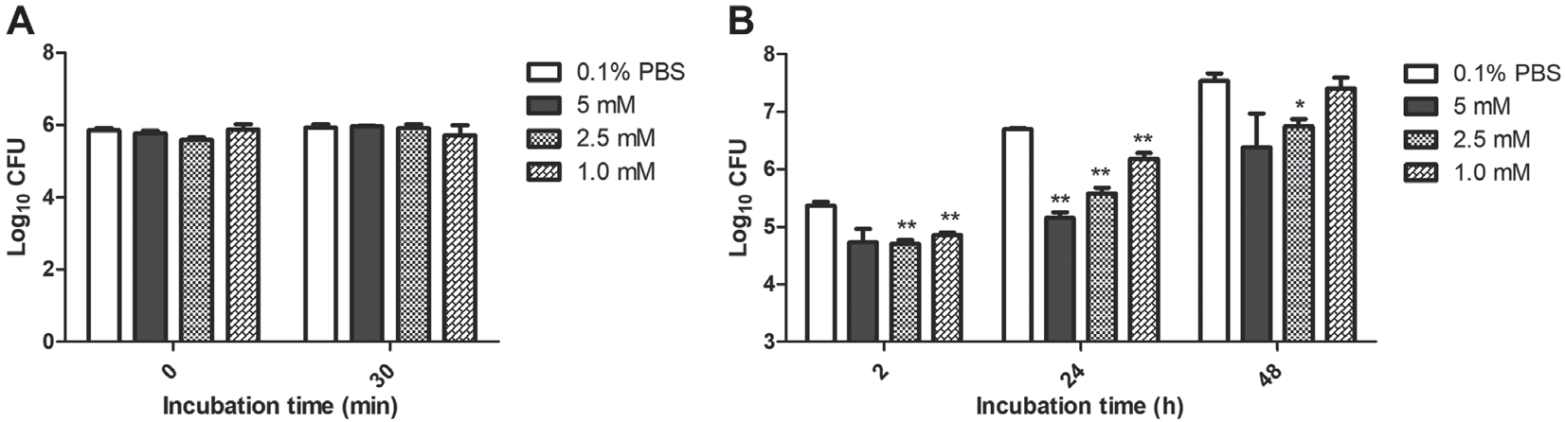
The effect of SP on *B. abortus* infection in macrophages. *Brucella* invasion was determined (**A**) and intracellular growth within macrophages (**B**) were evaluated at each time point. Three non-cytotoxic concentrations of SP (1, 2.5 and 5 mM) were used based on the results of cell viability and bactericidal assay. The data are represented as the mean ± SD of duplicate samples from at least three independent experiments. Statistically significant differences compared to control group are indicated by asterisk (*p < 0.05, **p < 0.01).

To find out the effect of SP on *B. abortus* infection in macrophage, cells were pretreated with SP for 12 h. CFUs were obtained and used to analyze the results. We found that there were no differences in internalization of *Brucella* into macrophages between SP treatment and 0.1% PBS as control at all time points tested (Fig. 2A). On the other hand, the SP-treated macrophages showed a significant reduction in intracellular growth at 2 h (**p < 0.01 at 1 and 2.5 mM), 24 h (**p < 0.01 at all concentrations) and 48 h (*p < 0.5 at 2.5 mM) post-infection (Fig. 2B). Furthermore, the reduction was observed in a dose-dependent manner. These findings showed that SP treatment could negatively affect the intracellular growth of *Brucella* in macrophages. Based on this result, we proceeded to determine which pathway is involved in macrophages treated with SP during *B. abortus* infection.

### Effect of SP on the Expression of Cytokines

**Fig. 3:**
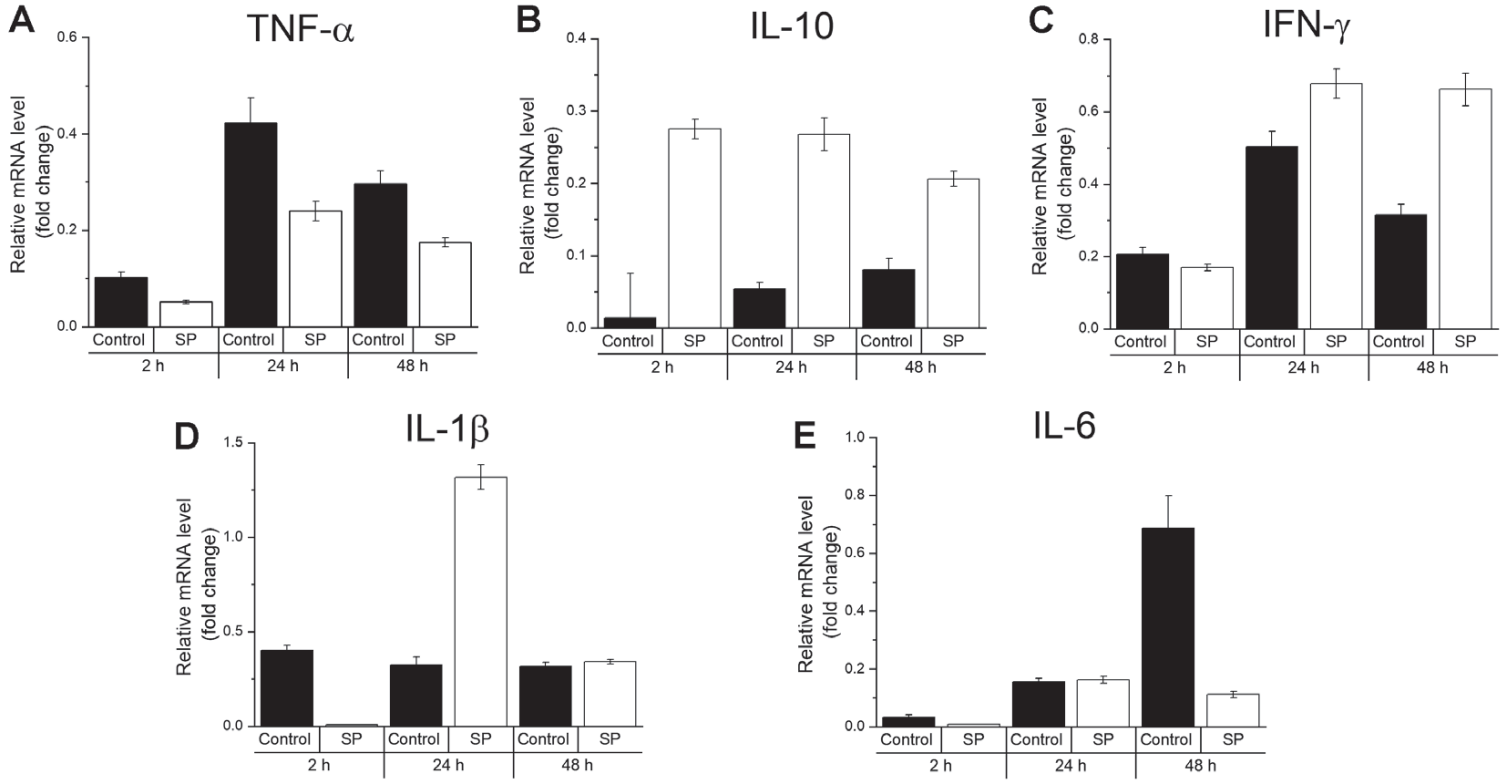
Effect of SP on the expression of cytokines mRNA in macrophages. The expression of inflammatory cytokines was measured at 2, 24 and 48 h post-infection. Level of inflammatory cytokines include TNF-α (**A**), IL-10 (**B**), IFN-γ (**C**), IL-1β (**D**) and IL-6 (**E**). Each cytokine mRNA was evaluated at least three times. The data are presented as the means ± SD for each group.

To identify the changes in the expression of cytokines from SP-treated macrophages, treatment, and infection were proceeded the same as that of the intracellular growth assay, and RNA was extracted from the cells for qRT-PCR. Here, we checked the expression of the five cytokines: TNF-α, IL-10, IFN-γ, IL-1β, and IL-6. Consequently, SP-treated macrophages showed a reduced expression of TNF-α at all time points (Fig. 3A). Conversely, SP-treated cells increased the expression of IL-10 at all time points (Fig. 3B). For IFN- γ, SP treatment induced the expression of these cytokines at 24 h and 48 h time point, and for IL-1β, the value was increased at 24 h time point, representing repression at 2 h and seems even at 48 h time point (Figs. 3C and 3D). Furthermore, for IL-6 expression, SP-treated macrophages inhibited IL-6 expression at 48 h, presenting similar levels at 2 and 24 h compared to the control (Fig. 3E). The results indicated that SP treatment could affect the expression of several cytokines that play an important role during *Brucella* infection in macrophages.

### Effect of SP on NF-κB Pathway

**Fig. 4:**
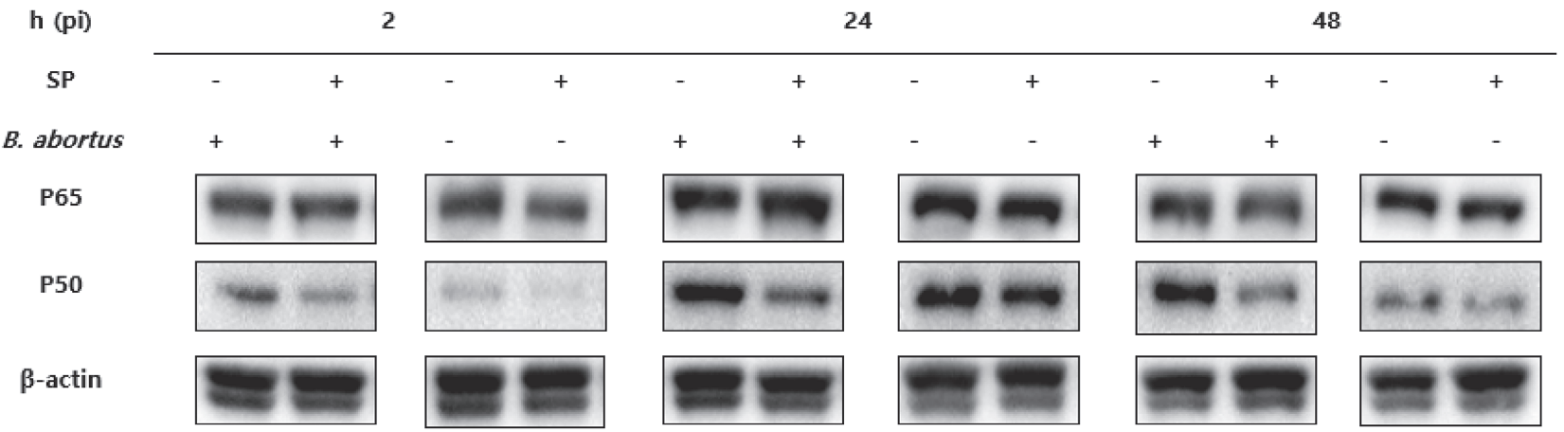
Effect of SP on phosphorylation of NF-κB in Raw 264.7 cells infected with *B. abortus*. Protocols on SP treatment and infection was done as that of the intracellular growth assay. The total protein was extracted for western blot analysis. The production of representative proteins was at 2, 24 and 48 h post-incubation were presented. The experiment was done in duplicates at least two times.

Several studies reported that SCFAs have an effect on inflammatory reaction relative to the NF-κB pathway. In fact, Inan *et al*. [10] studied that butyrate have a prominent anti-inflammatory effect in colitis, suppressing the NF-κB activity, while Liu *et al*. [11] established that acetate could prevent LPS-induced NF-κB protein 65 translocation to the nucleus. In accordance with these previous studies, we determined the involvement of NF-κB in controlling *B. abortus* in macrophages using western blot assay. We found that SP has a modest effect on phosphorylation of p65 (Fig. 4). However, SP treatment showed diminished phosphorylation of p50 at all time points regardless of infection (Fig. 4). Taken together, the results in the present study suggested that SP treatment could inhibit the phosphorylation of NF-κB.

### Effect of SP on ICR Mice

**Fig. 5:**
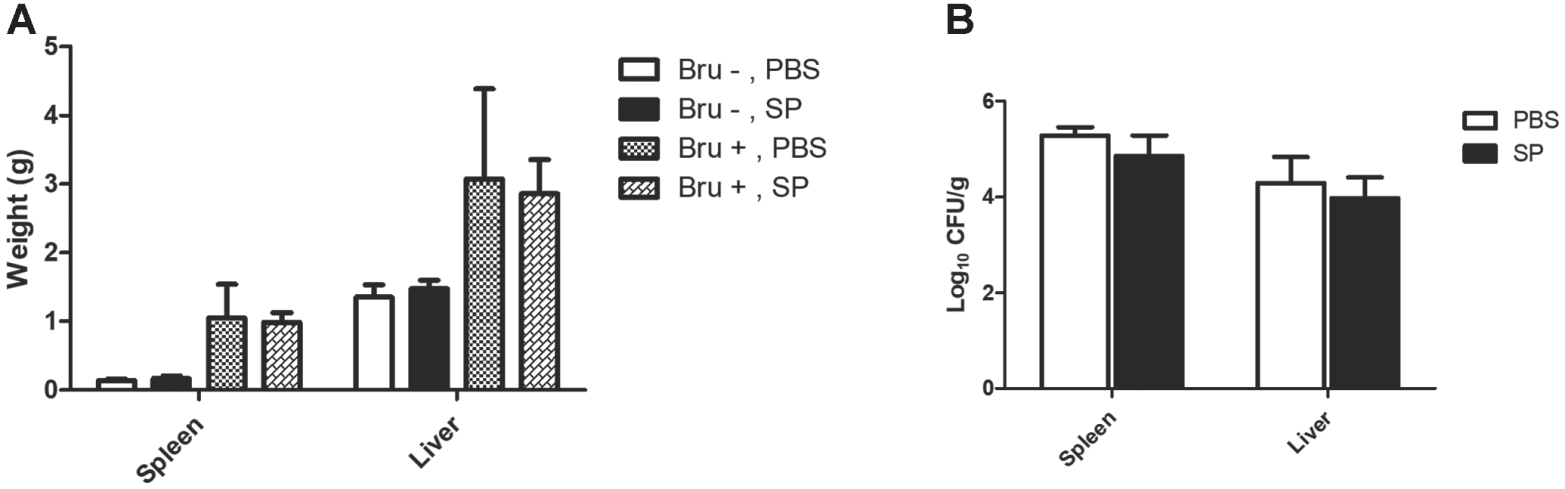
Effect of SP on bacterial proliferation in spleens and livers of *B. abortus*-infected mice. ICR mice were treated with SP or PBS orally for 5 days before infection and 14 days after infection. Mice were then sacrificed and spleens and livers were collected and weighed and a part was homogenized or used for histopathological experiment. The weight of spleen and liver (**A**), and the CFUs were counted from each organ (**B**).

At 14 days post-infection, the total weight of the spleens and livers was measured, and CFUs were determined. In addition, histopathological features such as necrosis, periportal inflammation, and microgranuloma in spleen and liver samples were estimated and scored according to the standard mentioned after H&E staining. The results indicated that the weight and the number of CFUs in the organs of the treatment group were slightly decreased compared to the control group (Figs. 5A and 5B). However, the total area of histopathological features resembled the same between SP treatment and non-treatment groups, representing similar scores in both spleen and liver (Data are not shown).

## Discussion

Macrophage is known as the first line of innate immunity and one of the major cells contributing to eliminate the foreign body by phagocytosis in a host. *Brucella* spp. are intracellular pathogens and the main target cell is macrophage. Hence the invasion and adaptation inside macrophages are regarded as key determinant for chronic brucellosis [12]. To survive in a host cell, *Brucella* have to reorganize the intracellular environment and minimize the effect of the host immune response interfering with brucellosis. However, what kind of mechanism the pathogens use and how these microorganisms survive in a host are not yet completely elucidated. Thus, interfering or interrupting mechanisms involved during *Brucella* infection or improving host immunity could be the successful way to eliminate brucellosis.

Several studies suggested that SP treatment enhances the antibacterial effect in a host. In a study done by Jeong *et al*. [13] SP ameliorates methicillin-resistant *Staphylococcus aureus* (MRSA) skin infection by attenuating bacterial growth. In another study conducted by Guinan *et al*. [14] SCFAs, including SP, inhibit the growth, germ tube, hyphae, and biofilm development of *Candida albicans* in vitro. Furthermore, SP treatment was reported to increase the bactericidal mechanisms including lysozyme gene expression and lysozyme enzyme activity in the Turbot head kidney macrophages, resulted in a reduction in intracellular *Edwardsiella tarda* survival [15]. Based on these previous studies, we investigated the effect of SP treatment on *Brucella* infection, and here we showed that SP treatment decreased intracellular growth in Raw 264.7 cells in a dose-dependent manner. To investigate how the intracellular environment is changed in macrophages treated with SP, we checked the expression level of five different cytokines including pro- and anti-inflammatory cytokines related to SP. Cytokines are known to participate in the process of *Brucella* infection and several studies demonstrated that SCFAs could inhibit the expression of pro-inflammatory cytokines whereas increase the expression of anti-inflammatory cytokines [16]. In this study, one of the major anti-inflammatory cytokines, IL-10, was increased at all time points. However, in contrast to what was postulated, not all pro-inflammatory cytokines showed to decrease. Here we showed that expression of TNF-α was inhibited at all time points while IL-6 was decreased at 48 h post-infection only compared to the control. Furthermore, IL-1β was higher even at 24 h, and IFN-γ was higher at 24 h and 48 h than the control group. Some investigators found that depending on the dose of the SCFAs and the particular cell type, these reagents may not impede immune responses and could even be stimulatory [17]. According to a study by Silva *et al*. [18] SCFAs, including SP, did not inhibit the overall pro-inflammatory gene expression. In other words, macrophage activation and induction of antimicrobial mechanisms by cytokines is complex and the expression mechanism and the effect of each cytokine varies accordingly [19]. Thus, SP treatment may influence the expression of cytokines and attribute to the anti-Brucella effect, but the properties of each cytokine could be different, and the individual role during infection needs further research.

Previous studies also reported that NF-κB regulated the expression of several genes involved in the inflammatory response and demonstrated that inhibition of NF-κB activation may be involved in the effects of SCFAs in immune cells [18-20]. Therefore, our hypothesis is that SP modulates transcription factors such as NF-κB and consequently have an effect on the expression of genes related to the inflammatory responses during *Brucella* infection. First, we observed inhibition of the constitutive p50 level in Raw 264.7 cells treated with SP compared to the control group. This tendency appeared similar at all time points and non-infection group. However, no difference in the activation of p65 was presented between SP treatment and control. Since the inflammatory process in *Brucella* infection is mainly sustained by macrophage-derived cytokines and the NF-κB is one of the main ways that modulate the expression of cytokines, the observed changes of cytokines expression due to SP treatment may at least be mediated by an inhibition of NF-κB activation in part. However, the exact mechanism by which SP restrain NF-κB activation in a host cell is still unclear in spite of a possible assumption. Recently, histone deacetylase (HDAC) inhibitors modulated the activity of NF-κB in a variety of cell types including macrophages and SP as one of the major SCFAs can act as an inhibitor of HDAC [18, 21-23]. A study done by Wang *et al*. [8] demonstrated that SP modulates inflammatory responses via inhibition of HDAC which attenuates LPS-induced NF-κB activation and inflammatory cytokine production in turn. According to previous studies mentioned earlier, we assume that SP has an effect on NF-κB as an inhibitor of HDAC since not all pro-inflammatory cytokines were inhibited (even IL-1β and IFN-γ increased). In conclusion, although the exact mechanisms of SCFAs remain to be studied further, the results in this study suggested that SCFA-induced immunological effects involved NF-κB-mediated signaling.

Another key question in this study is whether SP could have an effect in animals, hence so we applied SP treatment in mice for 19 days. According to histopathologic results, the concentration of SP we used in oral administration is non-toxic. However, our results demonstrated that oral administration of SP treatment was not significantly effective in mice contrary to in vitro findings. The weight and number of CFUs from the spleen and liver are reduced slightly compared to the one treated with PBS but not significant. Furthermore, the histopathological score of both organs was similar between SP treatment and non-treatment. SCFAs are extremely unstable with a high diffusion rate, and maintenance of a sufficient concentration of SP at a local site seems a major challenge to achieve their ideal effect in vivo. The study done by Cox *et al*. [7] showed that SCFAs induced immunological response related to prostaglandin E2 (PGE_2_) and cytokines in monocyte. Therefore, further mechanisms involved in a host and pharmacological methods are needed to confirm our in vitro findings with the in vivo model.

Taken together, this study suggested that SP inhibits intracellular growth of *Brucella* in macrophages by inhibiting NF-κB activation, which in turn changes the level of cytokines expression. Although further studies are needed to determine the exact mechanism involved, all the results supported the beneficial therapeutic potential of SP against *Brucella*, indicating the underlying molecular insights in changing inflammatory conditions to the host advantage.

## Figures and Tables

**Table 1:** List of primer sequences of cytokines for qRT-PCR.

Gene	Forward primer	Reverse primer
IL-10	5'-GGGTTGCCAAGCCTTATCGG-3'	5'-CTCTTCACCTGCTCCACTGC-3'
IL-6	5'-ACCACGGCCTTCCCTACTT-3'	5'-CATTTCCACGATTTCCCAGA-3'
IL-1β	5'-GGCAGGCAGTATCACTCATTGTGG-3'	5'-GCTCATGTCCTCATCCTGGAAGG
IFN-γ	5'-GTGGCATAGATGTGGAAG-3'	5'-GAGATAATCTGGCTCTGC-3'
TNF-α	5'-CAGGTTCTGTCCCTTTCACTCACT-3'	5'-GTTCAGTAGACAGAAGAGCGTGGT-3'
